# Aneurysmal Bone Cyst of the Distal Phalanx: A Case Report

**DOI:** 10.1016/j.jhsg.2026.101052

**Published:** 2026-09-10

**Authors:** Mehran Razavipour, Seyed Abolfazl Ghadiri, Soroosh Fateh

**Affiliations:** Orthopedic Surgery, Orthopedic Research Center, Mazandaran University of Medical Sciences, Sari, Iran

**Keywords:** Aneurysmal bone cyst, Lytic lesion, Tumor, Distal phalanx, Case report

## Abstract

Aneurysmal bone cysts (ABCs) are rare benign bone lesions, infrequently affecting the hand and exceptionally rare in the distal phalanx. Here, we present a unique case involving a woman aged 33 years who presented with progressive swelling and erythema of the distal phalanx of the right index finger. Magnetic resonance imaging demonstrated features suggestive of ABC, whereas plain radiographs were inconclusive. The patient underwent surgical excision and curettage through a volar approach, preserving the phalanx. Histopathologic examination confirmed the diagnosis, and no adjuvant therapy was administered. At the 4-year follow-up, the patient exhibited a full range of motion, no pain, and no signs of recurrence. To our knowledge, this is the first reported case of distal phalanx ABC in a finger treated with phalanx preservation rather than amputation. This case highlights the importance of including ABC in the differential diagnosis of hand tumors and supports the potential for conservative surgical management.

The distal phalanx of the fingers may be affected by a range of lesions that demonstrate an osteolytic appearance on radiographic imaging. The differential diagnosis includes primary and secondary bone tumors, metastatic disease, and inflammatory conditions. Among benign bone tumors of the hand, enchondromas are the most common and typically arise in the proximal phalanx. Phalangeal bones, particularly the distal phalanx, may also be affected by metastases originating from lung, breast, kidney, or prostate cancers. Other lytic lesions of the distal phalanx include giant cell tumor, giant cell reparative granuloma, glomus tumor, epidermoid inclusion cyst, brown tumor, and Ewing sarcoma.^1^

Aneurysmal bone cysts (ABCs) are rare, benign bone lesions, accounting for approximately 5% to 6% of all benign bone tumors. They primarily affect children and young adults, and most commonly involve the long bones. Approximately 70% occur as primary lesions, whereas 30% develop secondary to other bone pathologies, most frequently giant cell tumors.^2^ Hand involvement is uncommon, representing only 3% to 4% of cases, with the metacarpals affected more often than the phalanges.^3^

Management typically consists of curettage, with or without bone grafting, or resection of the distal phalanx.^4^ This report describes a rare case of an aneurysmal bone cyst involving the distal phalanx and emphasizes the importance of including ABC in the differential diagnosis of hand tumors. To the authors’ knowledge, this is the first reported case of an ABC of the distal phalanx treated with excision and preservation of the phalanx, rather than resection or amputation. The patient provided informed consent for publication. This case report was prepared in accordance with the CAse REport guidelines to ensure transparency, completeness, and standardized reporting.

## Case Presentation

A woman aged 33 years, right-handed and without a notable medical history, presented with progressive swelling and erythema of the distal phalanx of the right index finger, which worsened over 3 months. There was no reported history of trauma, systemic illness, or malignancy. Physical examination demonstrated mild tenderness and swelling on both the dorsal and volar aspects of the distal phalanx, without skin ulceration or nail deformity. Range of motion at the distal interphalangeal joint was mildly painful, particularly during flexion, with symptoms reported only recently. No axillary lymphadenopathy was observed.

**Figure 1:**
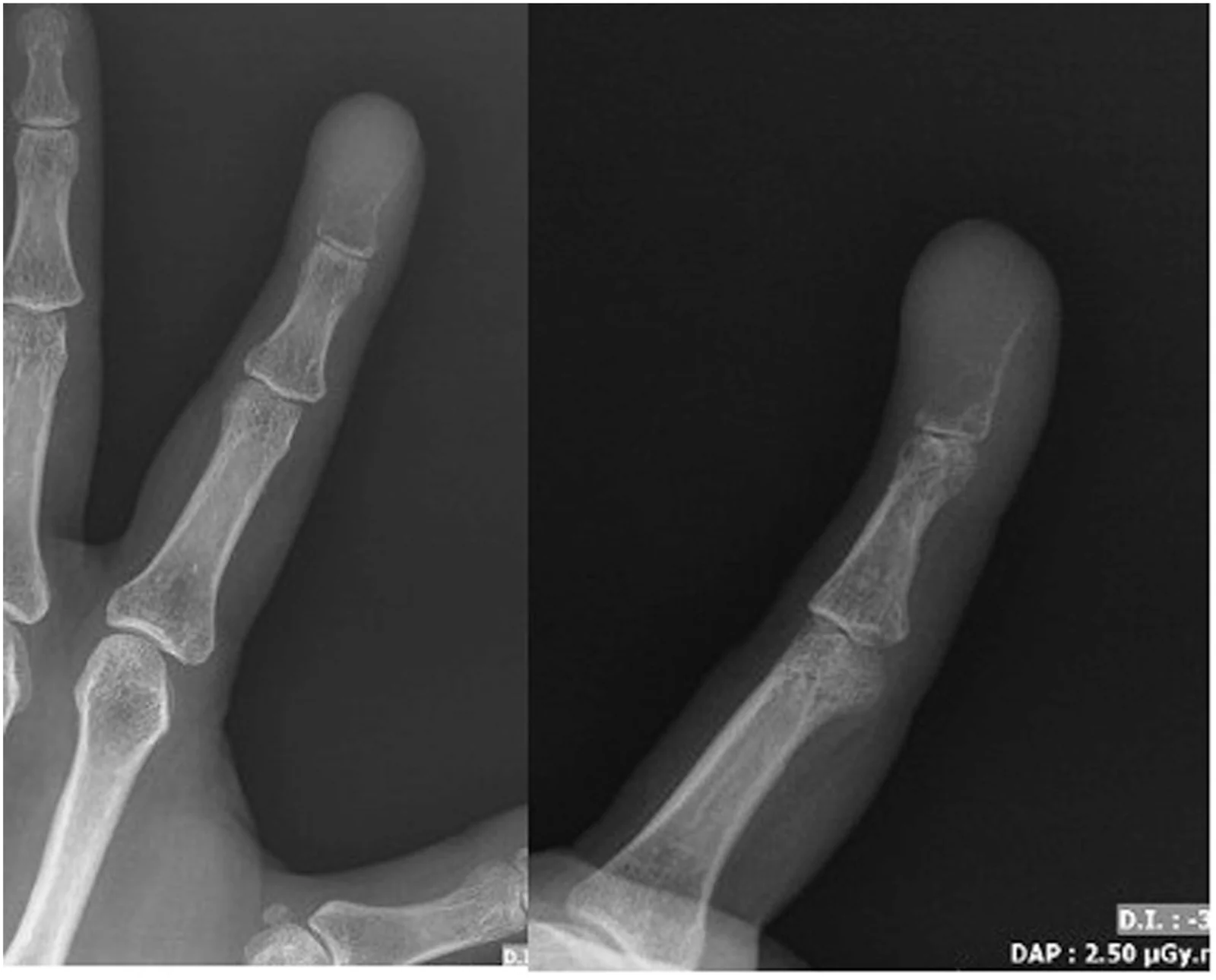
Plain x-ray. Demonstrating an expansile lytic lesion of the distal phalanx with dorsal cortex destruction.

**Figure 2:**
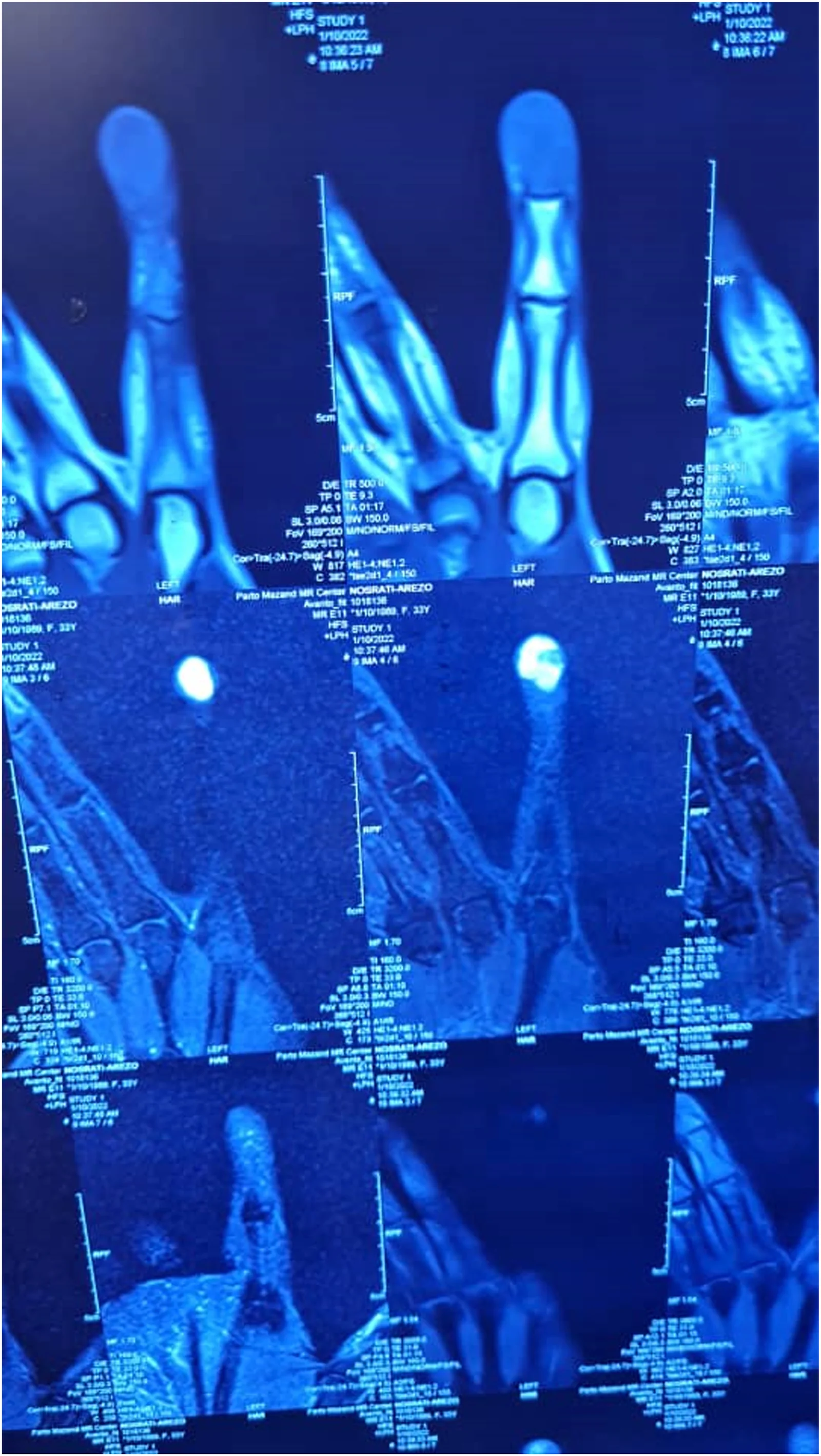
MRI. Characteristics of ABC.

Plain radiographs demonstrated a lytic, expansile lesion confined to the distal phalanx, without extension into the distal interphalangeal joint. Dorsal cortical destruction and volar cortical thinning were present, without internal calcifications or periosteal reaction (Fig. 1). Based on these findings, the differential diagnosis included giant cell reparative granuloma, aneurysmal bone cyst, epidermoid inclusion cyst, and malignant neoplasms. Magnetic resonance imaging (MRI) revealed a well-circumscribed lesion with internal septations, favoring a diagnosis of ABC (Fig. 2). Surgical excision and curettage were performed to obtain tissue for histopathological confirmation.

**Figure 3:**
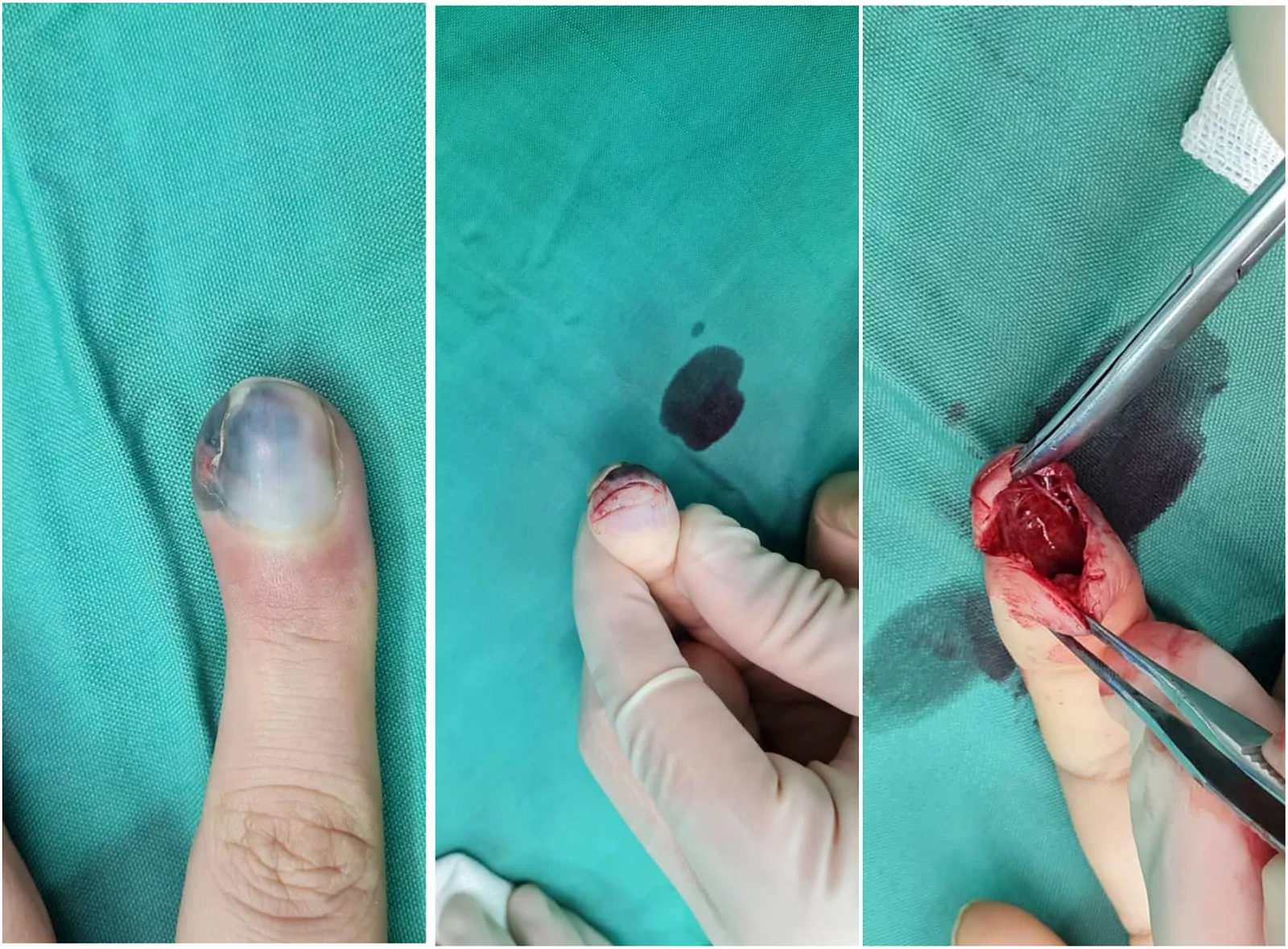
Intraoperative photos of the finger. Friable red tissue suggestive of ABC.

A volar approach was used to access the lesion. Intraoperatively, the tumor cavity contained bloody fluid and friable red tissue, consistent with the gross appearance of an ABC (Fig. 3). Complete excision was achieved through curettage, without bone grafting, and the distal phalanx was preserved. Tissue samples were submitted for histopathological examination to confirm the diagnosis.

**Figure 4:**
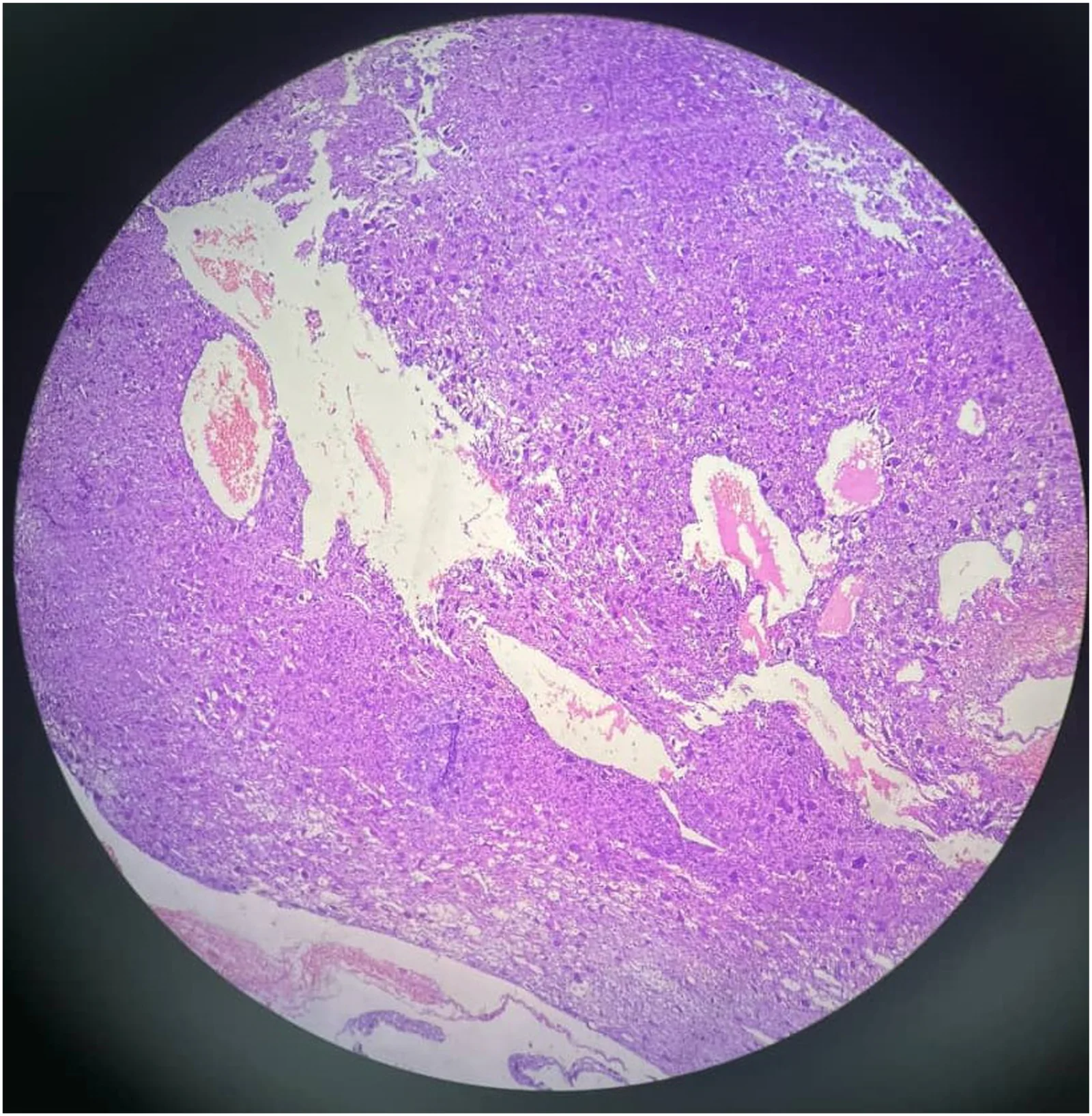
Pathologic view. Multiple blood lakes are surrounded by a giant cell-rich fibroblastic proliferation.

**Figure 5:**
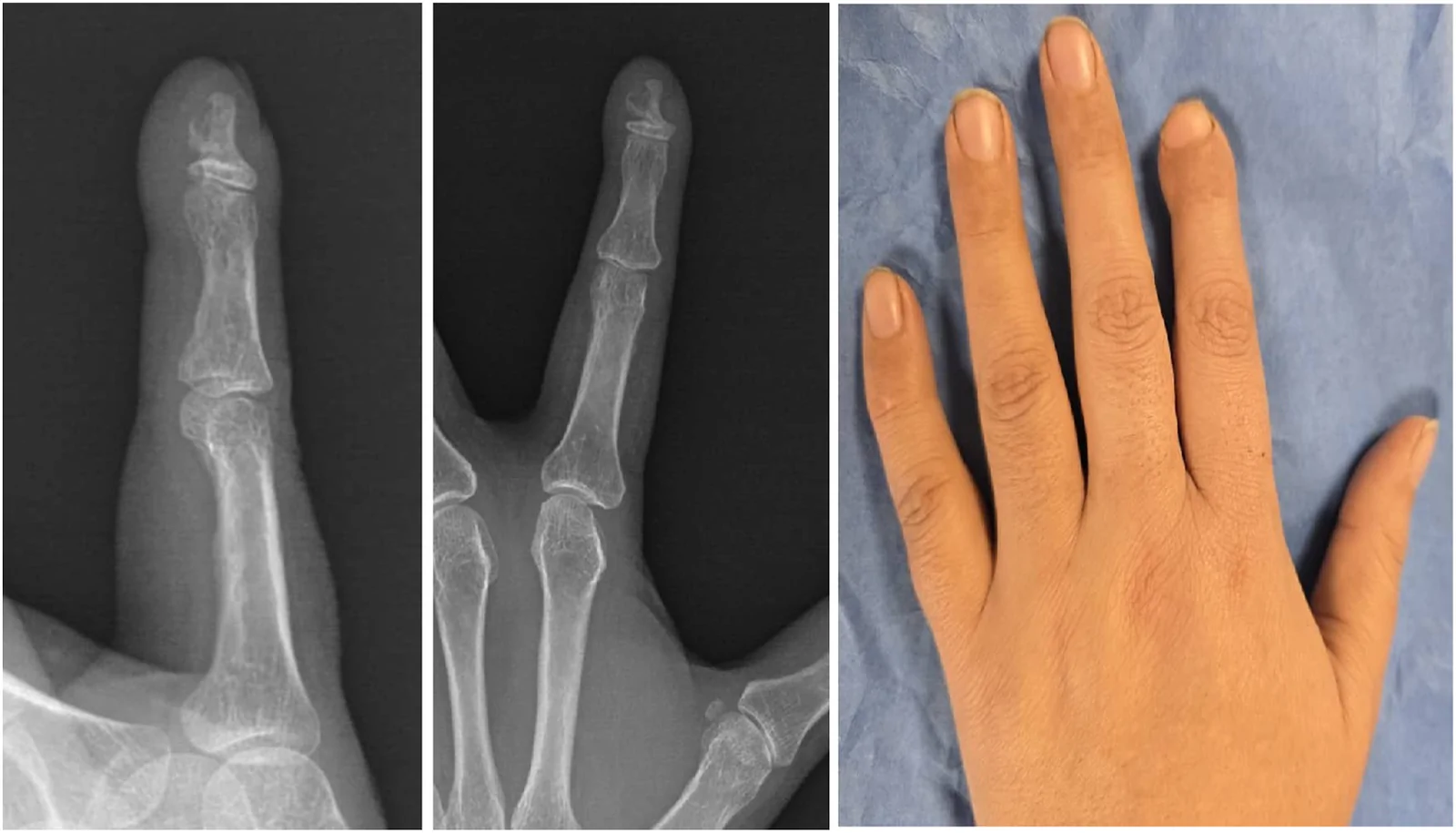
After 4 years of follow-up. Full functional recovery.

Histopathological analysis identified multiple blood-filled spaces surrounded by fibroblastic proliferation rich in giant cells, confirming the diagnosis of ABC (Fig. 4). No adjuvant therapy, including chemotherapy or radiotherapy, was required. The patient received standard postoperative wound care, and the skin healed without complications. At the 4-year follow-up, the patient had regained full function of the index finger, with no clinical or radiographic evidence of recurrence (Fig. 5).

## Discussion

ABCs are uncommon benign osseous lesions that most frequently arise in the long bones of the lower extremities.^4^ ABCs may occur as primary lesions or develop secondary to trauma, vascular malformations, or preexisting bone tumors, including osteoblastoma, giant cell tumor, osteosarcoma, chondroblastoma, nonossifying fibroma, or fibrous dysplasia.^5^ Hand involvement is rare and typically affects the metacarpals.^3^ Because of their rarity and overlapping radiographic features, ABCs in the hand are at risk for misdiagnosis. Accurate diagnosis requires integration of clinical, radiological, and histopathological findings.

ABCs most commonly present during the second decade of life.^3^ Presentation at age 33 years, as in this case, represents an uncommon late-onset ABC. Unlike metastatic or infectious processes, which often produce early pain, benign lesions such as ABCs typically present with painless swelling, with pain developing as the lesion advances.^1^ In this case, the initial clinical presentation included swelling and erythema of the distal phalanx, followed by gradual onset of pain and restricted range of motion over several months.

Lesions such as enchondroma, giant cell tumor, and metastatic disease more frequently involve the distal phalanx, making ABCs diagnostically challenging because of their rarity in this location.^1^ Radiographically, ABCs typically present as expansile, locally destructive lytic lesions with cortical thinning and occasional internal septation, whereas extension into adjacent soft tissue is uncommon. These imaging characteristics may overlap with those of other bone lesions, thereby expanding the differential diagnosis.^5^ On MRI, ABCs often appear as lobulated lesions with fluid–fluid levels; however, solid variants may present as heterogeneous expansile masses without these features.^6^ In the present case, plain radiographs were nondiagnostic, whereas MRI was suggestive of an ABC.

Treatment strategies for ABCs vary according to lesion size, location, and extent of osseous involvement. Management options include complete excision or curettage, with or without adjuvant therapy or bone grafting.^2^^,^^4^ Reports of ABCs involving the distal phalanx are scarce. In several published cases, resection of the phalanx with amputation at the level of the distal interphalangeal joint was performed.^6^, ^7^, ^8^ Two additional cases affecting the thumb were treated with curettage, one combined with burring and the other with curettage alone.^9^^,^^10^ Alternative treatments, including sclerosing agent injection, cryotherapy, radionuclide ablation, cryoablation, and arterial embolization, have been described for ABCs at other sites, sometimes in combination with curettage.^4^ However, none of these modalities has been reported for lesions of the distal phalanx.

Given diagnostic uncertainty and the limited size of both the lesion and the distal phalanx, surgical intervention was performed to obtain tissue for histopathological evaluation. The procedure involved complete excision of the tumor with meticulous curettage while preserving the remaining phalangeal structure to allow for further management if required after definitive diagnosis. A volar fingertip incision was used to access and excise the lesion. Intraoperatively, the presence of hemorrhagic fluid and friable red tissue was highly suggestive of an ABC^6^ and became the leading diagnostic consideration. Following histopathological confirmation of an ABC, no additional surgical treatment was indicated. The patient achieved an excellent outcome, with a full range of motion at the distal interphalangeal joint, absence of pain, and no cosmetic impairment.

Published literature indicates that ABCs of the hand most often involve the metacarpal and carpals.^3^ Only 5 cases of ABCs affecting the distal phalanx have been documented,^6^, ^7^, ^8^, ^9^, ^10^ 3 of which involved the thumb.^7^^,^^9^^,^^10^ The remaining 2 cases, both involving fingers, were treated with phalangeal amputation.^6^^,^^8^ The present case is unique as it represents the only reported instance of an ABC of the distal phalanx of a finger managed with preservation of the phalanx. At the 4-year follow-up, the patient remains asymptomatic, with no clinical evidence of recurrence, including swelling or discoloration, and no radiographic signs of bone expansion or lesion recurrence.

The outcomes in this case are promising; however, additional case reports and comparative studies are required to more fully evaluate treatment options for ABCs of the distal phalanx. Given the rarity of this presentation, cohort studies comparing surgical approaches and patient-specific variables would be valuable. Such investigations may help define optimal management strategies tailored to tumor characteristics and individual patient factors.

## Conflicts of Interest

No benefits in any form have been received or will be received related directly to this article.
